# Supplementary material for: Resolving the full spectrum of human genome variation using Linked-Reads
Source: Genome Res. 2019 Apr;29(4):635–45. doi: 10.1101/gr.234443.118 (PMC6442396; doi:10.1101/gr.234443.118)
Supplement: Supplemental Material [file supp_gr.234443.118_Supplemental_longranger-2.2.zip › longranger-2.2.tar.bz2!/lr2.2-public/lib/stan/cmdstan/stan/doc/api/html/math_2matrix_2multiply__lower__tri__self__transpose_8hpp_source.html]

Stan: src/stan/math/matrix/multiply\_lower\_tri\_self\_transpose.hpp Source File


|  |  |
| --- | --- |
|  | Stan  2.5.0  probability, sampling & optimization |


- Main Page
- Related Pages
- Namespaces
- Classes
- Files

- File List
- File Members

All Classes Namespaces Files Functions Variables Typedefs Enumerations Enumerator Friends Macros Pages

- src
- stan
- math
- matrix

multiply\_lower\_tri\_self\_transpose.hpp

Go to the documentation of this file.

1 #ifndef STAN\_\_MATH\_\_MATRIX\_\_MULTIPLY\_LOWER\_TRI\_SELF\_HPP

2 #define STAN\_\_MATH\_\_MATRIX\_\_MULTIPLY\_LOWER\_TRI\_SELF\_HPP

3

4 #include <stan/math/matrix/typedefs.hpp>

5

6 namespace stan {

7  namespace math {

8

17  inline matrix\_d

18  multiply\_lower\_tri\_self\_transpose(const matrix\_d& L) {

19  if (L.rows() == 0)

20  return matrix\_d(0,0);

21  if (L.rows() == 1) {

22  matrix\_d result(1,1);

23  result(0,0) = L(0,0) \* L(0,0);

24  return result;

25  }

26  // FIXME: write custom following agrad/matrix because can't get L\_tri into

27  // multiplication as no template support for tri \* tri

28  matrix\_d L\_tri = L.transpose().triangularView<Eigen::Upper>();

29  return L.triangularView<Eigen::Lower>() \* L\_tri;

30  }

31

32  }

33 }

34 #endif

typedefs.hpp

stan::math::matrix\_d

Eigen::Matrix< double, Eigen::Dynamic, Eigen::Dynamic > matrix\_d

Type for matrix of double values.

**Definition:** typedefs.hpp:23

stan::math::multiply\_lower\_tri\_self\_transpose

matrix\_d multiply\_lower\_tri\_self\_transpose(const matrix\_d &L)

Returns the result of multiplying the lower triangular portion of the input matrix by its own transpo...

**Definition:** multiply\_lower\_tri\_self\_transpose.hpp:18


---

[ Stan Home Page ]

*© 2011–2014,
Stan Development Team.*
